# Opposing Effects of Inhibitors of Aurora-A and EGFR in Autosomal-Dominant Polycystic Kidney Disease

**DOI:** 10.3389/fonc.2015.00228

**Published:** 2015-10-16

**Authors:** Anna S. Nikonova, Alexander Y. Deneka, Louisa Eckman, Meghan C. Kopp, Harvey H. Hensley, Brian L. Egleston, Erica A. Golemis

**Affiliations:** ^1^Program in Molecular Therapeutics, Fox Chase Cancer Center, Philadelphia, PA, USA; ^2^Kazan Federal University, Kazan, Russia; ^3^Cancer Biology, Drexel University College of Medicine, Philadelphia, PA, USA

**Keywords:** PKD1, Aurora-A kinase, mouse models, renal cyst, EGFR, SRC

## Abstract

Aurora-A kinase (AURKA) overexpression in numerous tumors induces aneuploidy, in part because of cytokinetic defects. Alisertib and other small-molecule inhibitors targeting AURKA are effective in some patients as monotherapies or combination therapies. Epidermal growth factor receptor (EGFR) pro-proliferative signaling activity is commonly elevated in cancer, and the EGFR inhibitor erlotinib is commonly used as a standard of care agent for cancer. An erlotinib/alisertib combination therapy is currently under assessment in clinical trials, following pre-clinical studies that indicated synergy of these drugs in cancer. We were interested in further exploring the activity of this drug combination. Beyond well-established functions for AURKA in mitotic progression, additional non-mitotic AURKA functions include control of ciliary stability and calcium signaling. Interestingly, alisertib exacerbates the disease phenotype in mouse models for autosomal-dominant polycystic kidney disease (ADPKD), a common inherited syndrome induced by aberrant signaling from PKD1 and PKD2, cilia-localized proteins that have calcium channel activity. EGFR is also more active in ADPKD, making erlotinib also of potential interest in this disease setting. In this study, we have explored the interaction of alisertib and erlotinib in an ADPKD model. These experiments indicated erlotinib-­restrained cystogenesis, opposing alisertib action. Erlotinib also interacted with alisertib to regulate proliferative signaling proteins, albeit in a complicated manner. Results suggest a nuanced role of AURKA signaling in different pathogenic conditions and inform the clinical use of AURKA inhibitors in cancer patients with comorbidities.

## Introduction

In its role as a mitotic regulator, Aurora-A kinase (AURKA) accumulates through G2 at the centrosome, becomes active at G2/M transition, and remains active through M phase as it translocates along the mitotic spindle to the midzone, with the bulk of AURKA degraded at the midbody at cytokinesis. A large number of proteins have been identified that directly associate with AURKA either in its N-terminal unstructured domain or C-terminal kinase domain, and regulate AURKA activation, including the highly studied TPX2 (1–7), but in addition, the scaffolding factors NEDD9, nucleophosmin/B23, PAK kinases, CEP192, and others (8–12). Human AURKA is overexpressed in many tumors arising from breast, colon, ovary, and other tissues, and to function as an oncogene when exogenously expressed in numerous cell line models (13–18). AURKA overexpression is invariably associated with increased number of centrosomes and multipolar spindles, which arise as consequence of failed cytokinesis, and reflect failure to downregulate AURKA activity at the end of mitosis. Inhibitors designed to block AURKA mitotic activity are currently undergoing clinical assessment as cancer therapeutics, with MLN8237/alisertib in multiple late-stage trials (19, 20).

Although most work on AURKA focuses on the activity of this protein in mitotic cells, a number of studies have now identified additional functions of AURKA in non-mitotic cells. For example, AURKA activity is required for neurite extension, in a post-mitotic cell population (21, 22). AURKA is also transiently activated by elevated cytoplasmic calcium, which triggers calmodulin binding to the N-terminal unstructured domain of AURKA and enhances binding to some partners, such as NEDD9, and reciprocally, AURKA phosphorylation of the polycystin 2 (PC2) calcium channel can inhibit its activity in interphase cells (23, 24). In addition, growth factor stimulation of quiescent ciliated cells induces NEDD9 expression and AURKA activation, leading to resorption of the cilium (25). These latter activities were of particular interest, as they not only potentially informed some roles of AURKA relevant to cancer (26, 27) but also connected AURKA activity to another pathological condition, autosomal-dominant polycystic kidney disease (ADPKD).

Autosomal-dominant polycystic kidney disease arises from inactivating mutations in the genes *PKD1* or *PKD2*, and currently has few treatment options (28). Formation of cysts is marked by multiple phenotypic changes in the cells lining renal tubules [reviewed in Ref. (28)]. These pleiotropic changes reflect the complex cellular action of the polycystins PC1 and PC2, products of the *PKD1* and *PKD2* genes. ADPKD is classified as a ciliopathy (29), based on the obligate functional heterodimerization of PC1 and PC2 on cell cilia, where PC1 normally acts as a flow sensor to trigger the calcium channel activity of PC2: calcium influx and other signaling interactions of the PC1/PC2 heterodimer act to restrain cell growth and govern the polarity of cell division in normal cells (30). Loss of cilia or defects in ciliary function can independently induce cyst formation (31).

As ADPKD signaling defects have become better understood, an unexpected feature has been the recognition that they possess extensive similarity to signaling defects seen in cancer (32). Exploiting these convergences, current research into the effective clinical management of ADPKD has been exploring the inhibition of signaling proteins, such as mTOR and SRC, that typically have elevated expression or activity in response to mutation of PC1/PC2 signaling, and actively contribute to cystic growth [reviewed in Ref. (30)]. Given the connections described above among AURKA, PC2, and cilia, and the identification that AURKA itself is elevated in cystic epithelia (23, 33), we previously explored efficacy of AURKA inhibition in controlling cyst growth in a mouse model of ADPKD (33). The initially surprising result of this study was that alisertib strongly exacerbated cyst formation. However, this outcome was compatible with an independent study that in the specific context of driver lesions in PKD1 or PKD2, genetic ablation of cilia reduces symptoms, suggesting the hypothesis that it is abnormal signaling rather than loss of signaling from the cilium that induces cyst formation (34). If so, then inhibiting signaling processes downstream of polycystins would potentially oppose the activity of alisertib. Epidermal growth factor receptor (EGFR) is activated in ADPKD (35, 36), and interacts with polycystins (37). In cancer, the combination of erlotinib and alisertib was first suggested by an siRNA screen that identified genes that influenced cellular response to inhibition of EGFR (38). In this work, AURKA inhibitors were shown to combine effectively with both small molecule and antibody inhibitors of EGFR *in vitro* and *in vivo*, providing the conceptual basis for two ongoing clinical trials (NCT01471964 and NCT01540682, clinicaltrials.gov). In the current study, to probe these novel actions of AURKA in ADPKD, we have evaluated the interaction of the EGFR inhibitor erlotinib with alisertib in control of cyst formation.

## Materials and Methods

### Mouse Strains and Drug Treatment

Conditional *Pkd1*^−/−^ mice in which tamoxifen induction of the Cre-flox regulatory system permits targeted inactivation of the Pkd1 gene *in vivo* have been described (33, 39, 40). *Pkd1fl/fl;Cre/Esr1*^+^ (referred to as *Pkd1*^−/−^), and control mice lacking an intact Cre-flox system (*Pkd1fl/fl;Cre/Esr1*^−^) mice were injected intraperitoneally with tamoxifen [250 mg/kg body weight (BW), formulated in corn oil] on post-natal days P2 and P3 for the early cyst induction, or post-natal days P35 and P36 for late cyst induction, to induce *Pkd1* deletion in the test group, as described (39). Alisertib (Millennium Pharmaceuticals, Inc., Cambridge, MA, USA) was formulated in 10% 2-hydroxypropyl-β-cyclodextrin (Sigma Aldrich, St. Louis, MO, USA) with 1% sodium bicarbonate and 20 mg/kg administered orally twice daily (BID), using a 5-day on/2-day off schedule. Erlotinib was formulated in 10% DMSO saline and 10 mg/kg administered orally once daily (QD), using a 5-day on/2-day off schedule. Treatment began at the age of 4 months and cyst growth monitored by magnetic resonance micro-imaging (MRI); mice were euthanized 10 weeks after the beginning of treatment to collect kidneys and liver for analysis. The Institutional Animal Care and Use Committee (IACUC) of Fox Chase Cancer Center approved all experiments involving mice.

### MRI Protocol and Image Analysis

Magnetic resonance micro-imaging was performed exactly as described in Ref. (33, 41, 42). Briefly, mice were anesthetized with 1–2% isoflurane in O_2_ and then imaged using a vertical bore 7-T magnet, Bruker DRX300 spectrometer, ParaVision 3.0.2 software (Bruker), and a single tuned ^1^H cylindrical radiofrequency coil. Kidney and cyst volume were quantified using Image J (43). For estimation of kidney volume, the kidney parenchyma was manually surrounded while excluding the renal pelvis, and summing up the products of area measurements of contiguous images and slice thickness, as in Ref. (44). Subsequently isolated kidney areas were prepared using defined settings for background subtraction and band passing, with a threshold set for each kidney based on the original images by targeting threshold values designating the transition between parenchyma and cyst at the border of the larger cysts in the kidneys. Cyst volume was estimated using a semi-automatic threshold approach (45, 46).

### Tissue Preparation and Histology

All tissues were collected and fixed in 10% phosphate-buffered formaldehyde (formalin) for 24–48 h, dehydrated and embedded in paraffin. Hematoxylin and eosin (H&E) stained 5 μm sections were used for morphological evaluation.

### Western Blotting

To analyze the expression levels of individual proteins, kidney tissues were lysed and resolved by SDS-PAGE. Western blotting was performed using standard procedures, and developed by chemiluminescence using Luminata Western HRP substrates (Classico, Crescendo and Forte) (EMD Millipore) and Immun-Star AP Substrate (Bio-Rad Laboratories). Quantification of signals on Western blots was done using the NIH ImageJ Imaging and Processing Analysis Software with signaling intensity normalized to loading control (β-actin or vinculin). Primary antibodies included anti-Src (Cell Signaling, #2110), anti-phospho-Src Tyr418 (Abcam, #ab4816), anti-S6 (Cell Signaling, #4858), anti-phospho-S6 S235/236 (Cell Signaling, #2317), anti-phospho-ERK Thr202/Tyr204 (Cell Signaling, #9101), anti-phospho-EGFR Y1068 (Cell Signaling, #3777), anti-phospho-EGFR Y1173 (Invitrogen, #44794G), anti-EGFR (Cell Signaling, #2646), anti-phospho-Akt S473 (Cell Signaling, #4060), anti-Akt (Cell Signaling, #2920), anti-Aurora-A (mouse, BD Transduction, #610939 and rabbit, Cell Signaling, #3092), anti-histone H3 (Cell Signaling, #3638S), anti-vinculin (Sigma, #V9131), and mouse anti-β-actin conjugated to HRP (Abcam, #ab49900). Secondary anti-mouse and anti-rabbit HRP-conjugated antibodies (GE Healthcare) were used at a dilution of 1:10,000 and secondary anti-mouse and anti-rabbit AP-conjugated antibodies (Jackson Immunoresearch Labs) were used at a dilution of 1:5,000.

### Phosphorylation Assay

Histone H3 (Upstate, Charlottesville, VA, USA) was used as substrate for AURKA kinase activity, using standard methods. Parallel aliquots without [γ32P]ATP were processed for SDS-PAGE. To assess Aurora-A activation, we performed an *in vitro* kinase assay using AURKA immunoprecipitated from whole kidney lysates using beads conjugated with anti-Aurora A antibody (Bethyl Laboratories, S300-070-3). Immunoprecipitation samples were incubated overnight with antibody at 4°C, washed, and resolved by SDS-PAGE.

### Statistical Analysis

Analyses were performed using STATA version 12. Data were analyzed using Wilcoxon rank-sum tests and generalized linear models with appropriate family and link functions (e.g., Gaussian or Gamma families with log or identity links). Where necessary, we estimated growth curves using generalized estimating equations (GEE) with exchangeable or Markov working correlation matrices to account for correlated data (46).

## Results

### Alisertib and Erlotinib Treatment of a Conditional Knockout Model for ADPKD: Modest Effect on Kidney Volume and Weight

**Figure 1:**
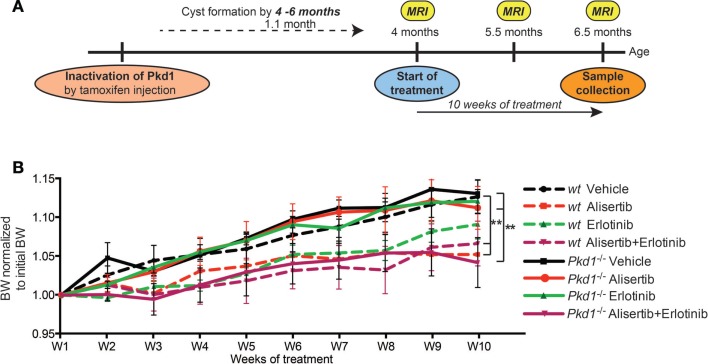
**Alisertib and erlotinib treatment of a conditional knockout model for ADPKD**. **(A)** Experimental design. *In vivo* experiments were performed using a conditional knockout mouse model for tamoxifen-induced, Cre-dependent ablation of *Pkd1* (39). **(B)** Body weight (BW) dynamics for the course of the treatment (vehicle, alisertib, erlotinib, alisertib + erlotinib), measured weekly. Differences between genotypes and drug treatment groups at 10 weeks were statistically significant between alisertib and alisertib + erlotinib versus the vehicle-treated groups in both wt and *Pkd1*^−/−^ groups. *P < 0.01 relative to vehicle.

We used a previously described *Pkd1* conditional knockout mouse model in which tamoxifen induction of a Cre-flox regulatory system allows targeted inactivation of the *Pkd1* gene *in vivo* (39, 40). In this system, the loss of *Pkd1* at post-natal day 28 results in development of renal cysts at ~4.5–5 months of age, progressing to severe enlargement of the kidney and renal failure at 6–7 months of age. The experimental outline is shown in Figure 1A. We defined four cohorts of *Pkd1*^−/−^ mice: Cohort 1 (*n* = 11), vehicle (10% cyclodextrin, 1% sodium hydrocarbonate, and 5% dextrose, with 10% DMSO mixed in 1:1 ratio) twice a day; Cohort 2 (*n* = 16), alisertib, 20 mg/kg, twice a day (40 mg/kg daily); Cohort 3 (*n* = 13), erlotinib, 10 mg/kg, once a day; and Cohort 4 (*n* = 14), alisertib 20 mg/kg, twice a day plus erlotinib, 10 mg/kg, once a day (2 h after the morning dose of alisertib). Parallel cohorts 5–8 were also run, with wild type mice that received the same dosing regimen: each of these cohorts contained 8–10 animals. Starting at the time of injection, mice were weighed weekly. Treatment with alisertib or alisertib plus erlotinib resulted in slower weight gain over 10 weeks in both wild type and *Pkd1*^−/−^ groups, while erlotinib alone had no effect on weight gain (Figure 1B).

**Figure 2:**
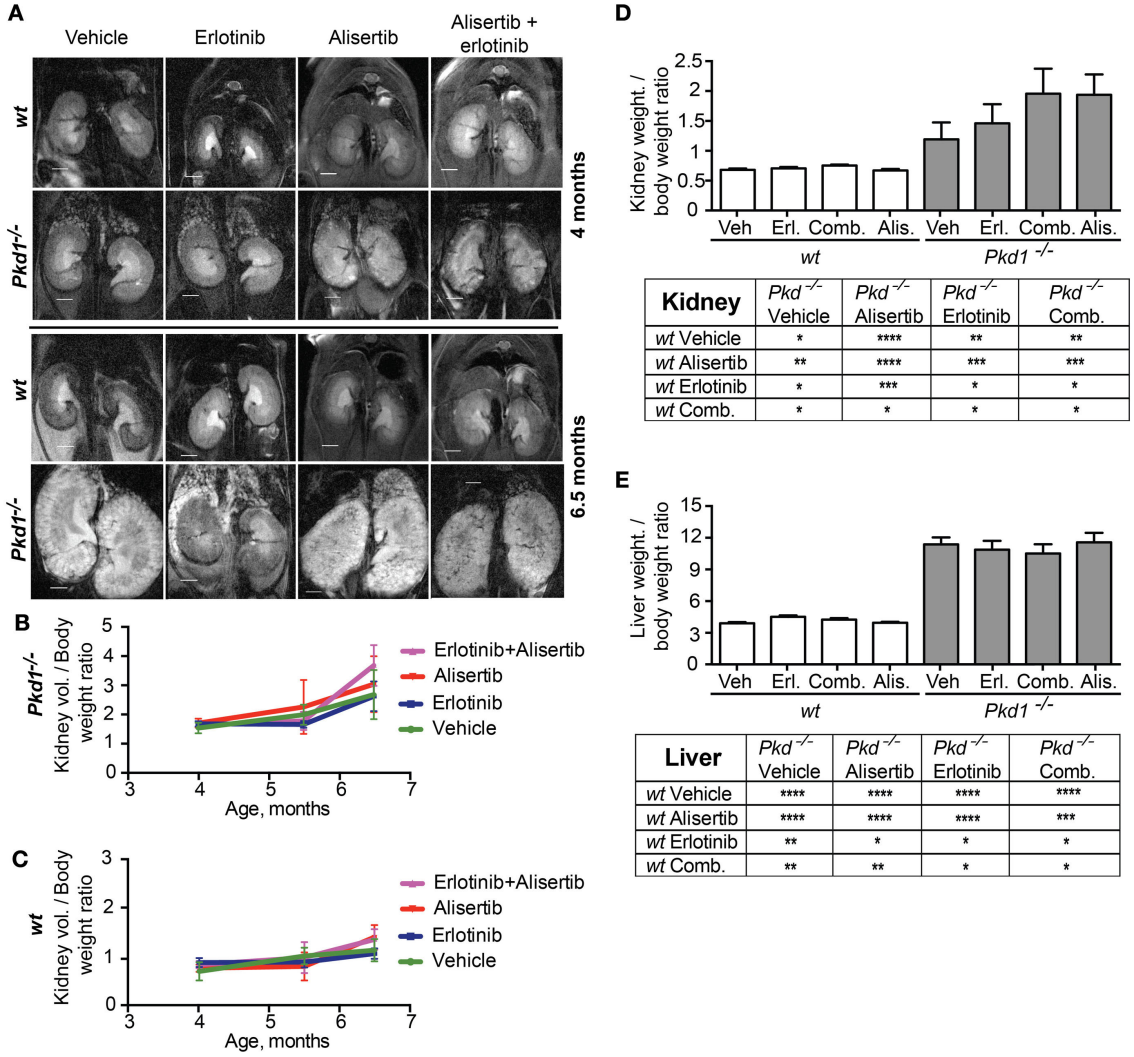
**Effects of alisertib and erlotinib on kidney growth**. **(A)** Representative images of murine kidneys acquired by using magnetic resonance micro-imaging (MRI) approach, at 4 (top) and 6.5 (bottom) months of age following treatment with vehicle, alisertib, erlotinib, and a combination of these drugs. **(B,C)** Quantification of the MRI imaging results for kidney volume, normalized to body weight for *Pkd1*^−/−^ **(B)** and wt **(C)** mice. Differences at experimental endpoint (10 weeks of treatment) are not statistically significant. **(D,E)** Direct measurement of kidney **(D)** and liver **(E)** weight normalized to body weight for *Pkd1*^−/−^ and wt mice at experimental endpoint (10 weeks of treatment). Top – graphs representing results; bottom – summary of *P*-values for the presented data. *P < 0.05, **P < 0.01, ***P < 0.001, and ****P < 0.0001.

For analysis of kidney enlargement over time, *Pkd1*^−/−^ and wt mice were assessed at 4, 5.5, and 6.5 months of age using a MRI approach (41) (Figure 2A). In general, drug effects on rate of kidney growth did not rise to statistical significance. Following normalization to BW, alisertib slightly increased the rate of kidney growth versus vehicle-treated *Pkd1*^−/−^ mice at all time points (Figure 2B). Erlotinib did not significantly affect growth, at all time periods. The alisertib/erlotinib combination initially resulted in a rate of kidney growth similar to vehicle or erlotinib-treated mice, but at latter time points, the ratio of kidney volume to BW indicated a phenotype more similar to alisertib. However, it is important to note that mice treated with this drug combination had a significantly lower BW (Figure 1B), which likely contributes to the difference. As a control, we established that no drug affected kidney volume increase in wild type mice (Figure 2C). After 10 weeks, mice were euthanized and kidney weight to BW ratio directly determined. This confirmed findings from MRI, with a non-statistically significantly trend toward elevated BW in *Pkd1*^−/−^ mice treated with alisertib or alisertib/erlotinib, and all mice with a *Pkd1*^−/−^ genotype having a statistically significant greater kidney weight than all wt mice (Figure 2D). Hepatic cysts are a common feature of ADPKD in humans, occurring in a significant number of patients. In a previous study, we showed that inhibition of HSP90 significantly reduced liver cyst burden (42), while also reducing the rate of development of kidney cysts (41). In the present study, the alisertib effect was specific to kidney tissue, and no effect was seen with any drug treatment in liver from wild type or *Pkd1*^−/−^ mice (Figure 2E).

### Alisertib and Erlotinib Treatment of a Conditional Knockout Model for ADPKD: Drug Interactions in Control of Cyst Development

**Figure 3:**
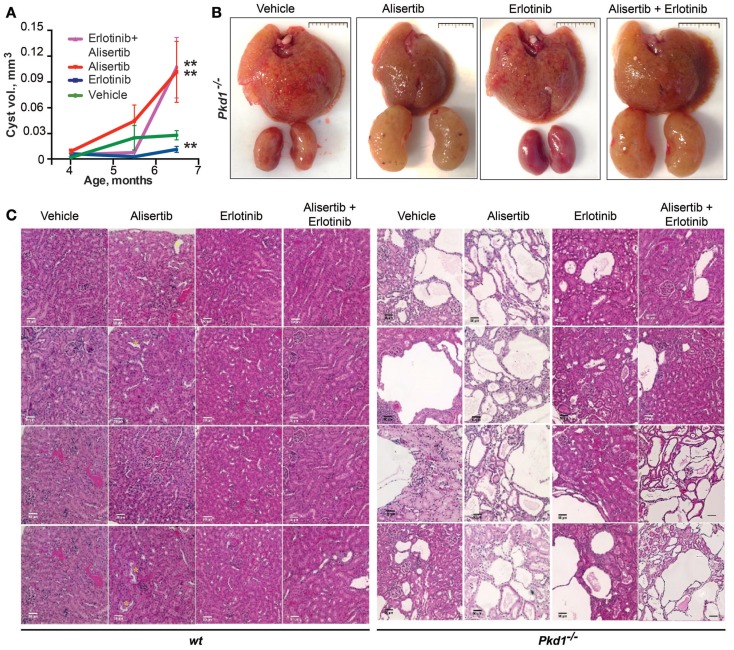
**Cystogenesis during drug treatment in *Pkd1*^−/−^ and wt mice**. **(A)** Ratio of renal cyst volume to body weight in *Pkd1*^−/−^ mice in the four drug treatment groups. *P < 0.05 and **P < 0.01 relative to vehicle treatment. **(B)** Representative images of collected livers (top) and kidneys (bottom) from each treatment cohort; scale bar, 1 cm. **(C)** Representative light microscopy images from H&E slides, reflecting cystic burden of *Pkd1*^−/−^ and wt mice in all treatment groups at experimental endpoint (10 weeks of treatment). Images taken at 20× magnification; scale bar, 50 μm.

The development of cysts was analyzed by quantification of MRI imaging (Figures 2A and 3A), and subsequently confirmed by visual assessment of hematoxylin and eosin (H&E) stained tissues collected after 10 weeks of treatment (Figures 3B,C). No wild type mice developed cysts. Among the *Pkd1*^−/−^ mice, there was some heterogeneity in cyst development between individual animals, in concordance with the basic biology of the disease and previous reports using the model (39–41). Notably, the erlotinib treatment strikingly reduced cystogenesis in most animals, at all time points, in a statistically significant effect (Figure 3A). Alisertib treatment elevated cyst growth early, and cystogenesis was much greater than in vehicle-treated animals by the experimental endpoint, as previously noted (33). Interestingly, the alisertib/erlotinib combination treatment caused an initial delay in the formation of cysts, similar to erlotinib. However, at later time points, the beneficial effect was lost, and at experimental endpoint, the overall phenotype resembled alisertib-treated mice. Mice treated with alisertib or erlotinib plus alisertib had an extremely heterogeneous phenotype at the experimental endpoint. Although the majority had highly cystic kidneys, some had only limited cysts, suggesting a stochastic effect in response to drug between individual animals, and accounting for the large error bars.

### Signaling Consequences of Alisertib and Erlotinib Treatment *Pkd1*^−/−^ and wt Kidneys

To better understand the functional interaction of inhibition of AURKA and EGFR, we analyzed activation of the signaling of these drug targets and of signaling pathways relevant to ADPKD in kidney tissue collected from *Pkd1*^−/−^ and wt mice after 10 weeks of drug treatment.

**Figure 4:**
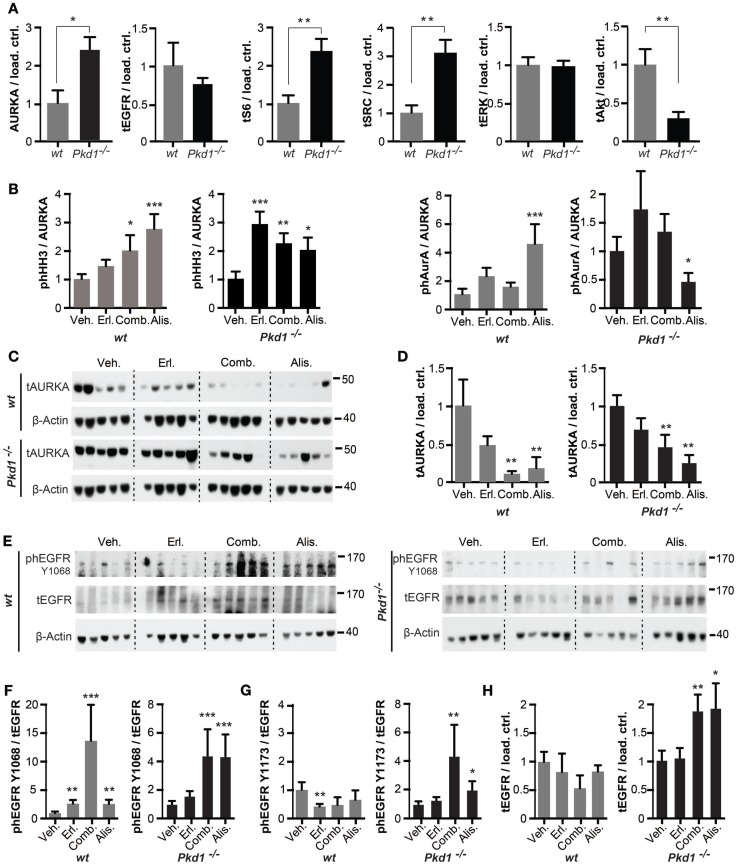
**Drug inhibition of targets in *Pkd1*^−/−^ and wt mice**. **(A)** Quantification of Western data for indicated proteins from kidney lysates prepared from vehicle-treated wt or *Pkd1*^−/−^ mice, normalized to β-actin or vinculin-loading control. *P < 0.05 and **P < 0.01 relative to vehicle treated. **(B)** Aurora-A (AURKA) was immunoprecipitated from kidney lysates and used for *in vitro* kinase with γ-32P-ATP to indicate autophosphorylation and phosphorylation of histone H3 kinase (HH3) (top two rows), with parallel blots probed by Western to allow normalization to total AURKA and HH3 in reaction. Quantitation of data from complete cohort of mice in each treatment group, indicating ratio of phosphorylated HH3 or AURKA to total immunoprecipitated AURKA in *wt* and *Pkd1*^−/−^ mice following indicated drug treatments. *P < 0.05, **P < 0.01, and ***P < 0.001 relative to vehicle treated. **(C)** Western analysis showing representative expression of total Aurora-A (tAURKA) in kidney lysates after 10 weeks of treatment with indicated drugs, with β-actin loading control. **(D)** Quantitation of data from complete cohort of mice in each treatment group, indicating ratio of total AURKA to β-actin in *wt* and *Pkd1*^−/−^ mice following indicated drug treatments. **P < 0.01 relative to vehicle treated. **(E)** Western blot with representative images of kidney lysates from each treatment cohort showing expression of EGFRphosphorylated (ph) at the indicated amino acids, total EGFR (tEGFR) or β-actin loading control. **(F–H)** Quantitation of data from complete cohort of mice in each treatment group, indicating ratio of phEGFR-Y1068 to tEGFR **(F)**, phEGFR-Y1173 to tEGFR **(G)**, or tEGFR to loading control **(H)**. *P < 0.05, **P < 0.01, and ***P < 0.001, relative to vehicle treated.

Under conditions of vehicle treatment, AURKA expression was elevated in *Pkd1*^−/−^ versus wt kidneys, as previously reported (33) (Figure 4A). *In vitro* kinase analysis of phosphorylation of the substrate histone H3 (HH3) by AURKA immunoprecipitated from kidney lysates, or the autophosphorylation of immunoprecipitated AURKA, normalized to total levels of immunoprecipitated kinase (Figure 4B), surprisingly indicated that drug treatments did not produce statistically significant effects on AURKA activity. However, parallel Western analysis (Figures 4C,D) indicated that total levels of AURKA were significantly depleted in tissue treated with each of the drugs, particularly in those treated with alisertib or alisertib plus erlotinib. Generally, similar effects of drug treatment were observed in wt kidneys. Hence, the primary consequence of alisertib treatment was to reduce overall AURKA activity by reducing total AURKA expression.

Epidermal growth factor receptor activation is reflected by phosphorylation of EGFR at Y1068, which allows it to associate with GRB2 to activate downstream signaling cascades (47), and at Y1173, which is important for SHC binding and EGFR internalization (48). Total EGFR expression was not elevated in wt versus *Pkd1*^−/−^ kidneys (Figure 4A). However, in *Pkd1*^−/−^ kidneys, Y1068 phosphorylation was significantly increased by treatment with alisertib, or alisertib plus erlotinib, and Y1173 phosphorylation was increased, albeit to a lesser degree (Figures 4E–G). Furthermore, in *Pkd1*^−/−^ kidneys, total EGFR expression was also elevated by treatment with alisertib or the alisertib/erlotinib combination (Figures 4E,H). Together, these results emphasized the role of AURKA inhibition in potentiating proliferative signaling relevant to a severe cystic phenotype. By contrast, no drug treatment significantly affected EGFR expression or phosphorylation on Y1173 in relation to vehicle in wt kidneys, although interestingly, the alisertib/erlotinib combination significantly induced Y1068 phosphorylation in a subset of wt kidneys (Figures 4E–H).

**Figure 5:**
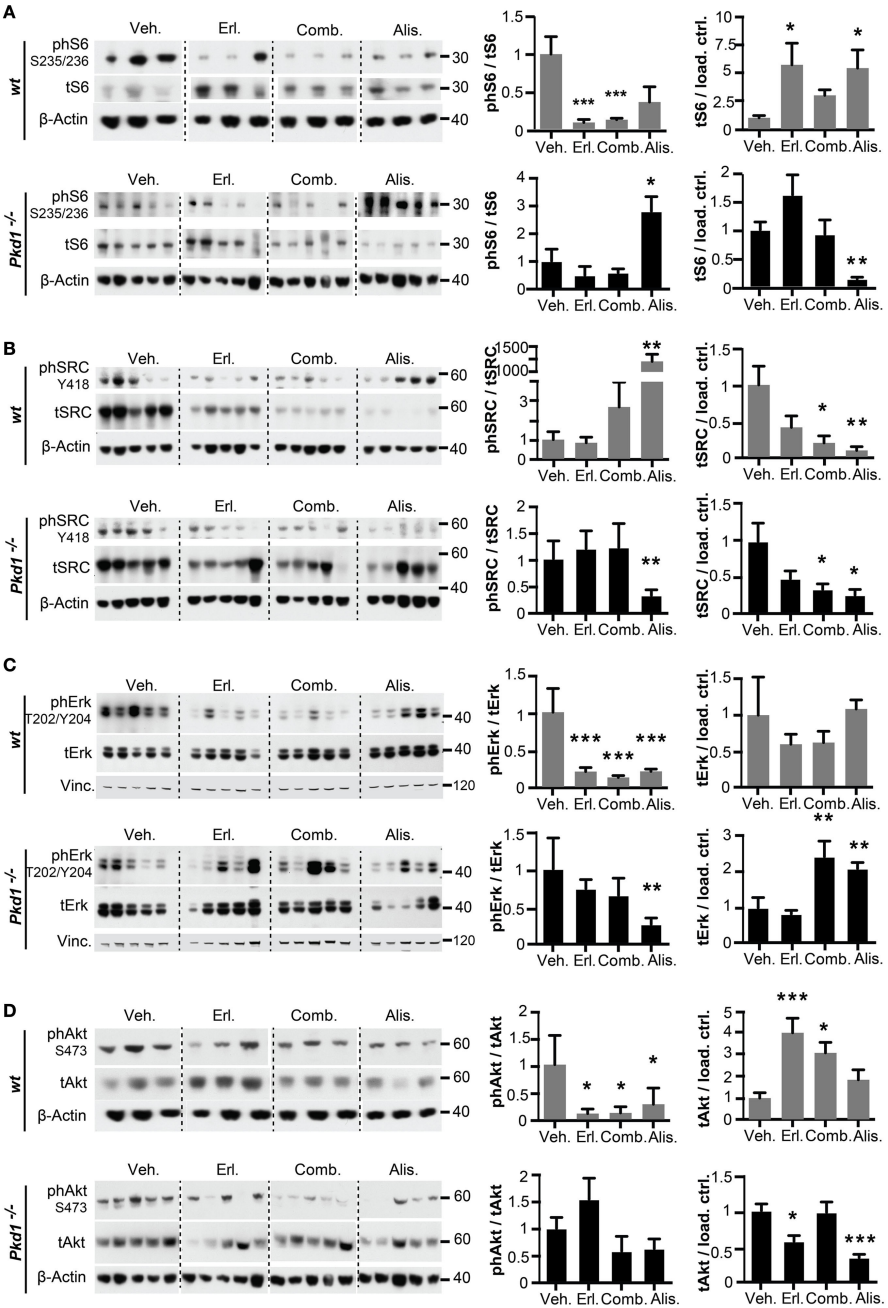
**Drug inhibition of ADPKD-related signaling proteins in *Pkd1*^−/−^ and wt mice**. S6 **(A)**, SRC **(B)**, ERK1/2 **(C)**, and AKT **(D)** in kidney lysates after 10 weeks of treatment with indicated drugs. Western analysis (left) showing representative expression of phosphorylated and total expression of proteins and quantitation of data (right) from complete cohort of mice in each treatment group with analysis, as described in Figure 4. **(A–D)** Ratios of phosphorylated to total protein for S6 **(A)**, SRC **(B)**, ERK1/2 **(C)**, and AKT **(D)** are shown in the left graph, and ratios of total protein to β-actin or vinculin (vinc) loading control are shown in the right graph. *P < 0.05, **P < 0.01, and ***P < 0.001 relative to vehicle treated.

We then analyzed the activity and expression of the ADPKD-related proteins S6, SRC, ERK1/2, and AKT (Figures 4 and 5). For S6, AKT, and SRC, total levels were elevated in *Pkd1*^−/−^ versus wt kidneys (Figure 4A). The patterns of response to drug treatment were complicated for these downstream pro-proliferative proteins. Focusing first on alisertib in *Pkd1*^−/−^ kidneys, this treatment significantly reduced levels of total S6 and SRC, and increased total levels of ERK1/2 (Figure 5). However, alisertib also resulted in a very significant increase in the ratio of active (phosphorylated) S6, leading to a net gain in S6 activity in kidney lysates, compatible with an increased cystic phenotype. By contrast, activity of SRC and ERK was reduced by alisertib in *Pkd1*^−/−^ kidneys. These patterns of expression and activation were very different from those evoked by alisertib treatment of wt kidneys. In wt kidneys, alisertib very significantly reduced S6 and ERK1/2 activation, and reduced SRC expression. Alisertib treatment also resulted in a variable pattern of SRC activation, with three mice having very high levels of phosphorylated SRC, but most having SRC activity reduced. Whereas erlotinib or erlotinib plus alisertib effectively reduced ERK1/2 and S6 activity in wt kidneys, these treatments were less effective in *Pkd1*^−/−^ kidneys. With the exception of effect on total ERK1/2 expression in *Pkd1*^−/−^ kidneys, erlotinib, and erlotinib plus alisertib resulted in statistically non-distinct effects on the expression and activation of the signaling proteins analyzed. This was surprising, given the very different results of these treatments on cystic phenotype.

## Discussion

As significant findings, this study has established that the cystogenic activity of the AURKA inhibitor in ADPKD can be partially reversed by treatment with erlotinib, and for the first time showed that erlotinib itself has a potent activity in limiting cystic growth. Second, it also demonstrated that erlotinib and alisertib elicited distinct response profiles in the kidneys of wt versus *Pkd1*^−/−^ mice, potentially reflecting differences in signaling landscape associated with the replacement of the normal renal parenchyma with cysts. Third, it also demonstrated that in the context of drug treatment, changes in degree of cystogenesis in *Pkd1*^−/−^ mice could not be aligned with specific changes in expression or activity of AKT, S6, ERK1/2, or SRC, in spite of the common association of elevated activity of these signaling proteins with disease etiology. Finally, as discussed below, this work emphasized some significant differences between the interactions of alisertib and erlotinib in the context of ADPKD versus cancer, such that this treatment is potentially beneficial in cancer but not ADPKD.

These findings confirmed earlier reports that AURKA activity was associated with cyst formation, and that inhibition of AURKA exacerbated cystogenesis in the context of genetic loss of *Pkd1* (23, 24, 33). A particularly interesting finding was that treatment with alisertib alone or in combination with erlotinib was more strongly associated with loss of AURKA protein, rather than inhibition of AURKA activity. There are two potential explanations for this observation. First, AURKA expression is highly regulated by protein degradation, and the protein is more susceptible to degradation when catalytically inactive (49, 50). Hence, alisertib treatment may be elevating the rate of AURKA destruction, and in this context, the elevated activity of the remaining AURKA may represent a sub-population effectively protected by interaction partners (49, 50). Second, AURKA expression has been reported as most abundant and active in the renal epithelia of early cysts (23, 24, 33). However, in the tissue isolated at the end of this experiment and used for signaling analysis, the majority of tissue in alisertib- and alisertib/erlotinib-treated cells reflects loss of normal renal structure and replacement with enlarged late-stage cysts that have in many cases lost epithelial lining, and fibrotic tissue. In this interpretation, a difference in tissue composition explains the loss of AURKA activity. Potentially, both explanations contribute to the observed phenotypes.

In analysis of signaling consequences of AURKA inhibition, alisertib reduction of SRC expression and activity was notable. One previous study has shown direct AURKA phosphorylation of SRC enhances SRC activity (51); it is possible that this phosphorylation also contributes to SRC stability, so that alisertib destabilizes SRC. The significant effect of AURKA inhibition on S6 phosphorylation is compatible with the enhanced cystogenesis seen in alisertib-treated mice. However, comparison of alisertib versus alisertib/erlotinib-treated mice confounds simple interpretation of specific signaling changes as relevant to the experimental endpoint, given the cystogenic phenotype of the combination resembles alisertib, whereas the signaling phenotype resembles erlotinib. This discordance suggests that while proteins such as S6 and SRC may have elevated expression and activity in ADPKD, their contribution to disease pathogenesis is not an essential driver of disease progression. This interpretation would be compatible with the limited activity of inhibitors of the S6 activator mTOR (52–54), or inhibitors of SRC (55), in assessment for treatment of polycystic kidney disease.

Although erlotinib has not previously been assessed, some prior studies have used alternative inhibitors of EGFR (56–58) or related ERBB-family kinases (59) in various models for cyst formation. Typically although not invariably, these studies have found inhibition of EGFR/HER2 signaling reduced cystic burden. In this study, we show that erlotinib is well tolerated and effective in controlling cyst growth in the context of loss of Pkd1, and that erlotinib can delay the growth of cysts that alisertib promotes. These results suggest that erlotinib may be useful for clinical evaluation in ADPKD. It is also interesting and important to note that erlotinib treatment reduces the level of AURKA in wt but not *Pkd1*^−/−^ kidneys (Figure 4). In this case, one possible explanation is that erlotinib treatment causes wt cells to accumulate in the G1 phase of cell cycle by inhibiting multiple mitogenic effector pathways (60), hence reducing the population of G2/M phase cells in which AURKA is most abundant, whereas this inhibition is partially overcome in ADPKD tissue. This interpretation would suggest that small pool of AURKA at the basal body of cilia (61) – the pool potentially most relevant to the control of cystic severity, based on the model developed by Ma and colleagues (34) – might not be affected by erlotinib, as it is active in G0/G1. Test of this idea requires development of antibodies or probes suitable for analysis of AURKA activity by immunofluorescence in mouse tissue, currently a technical limitation on performing this work (50). Additional analyses of future interest would be the profiling of renal function (rather than cystic burden) following treatment with alisertib, erlotinib, or the combination, as well as broader profiling of gene expression changes following such treatments.

Finally, understanding the basis of AURKA and EGFR activity in ADPKD is of considerable interest for the field of oncology. One in 500 individuals suffers from ADPKD, many of whom will ultimately develop cancer and could potentially be treated with alisertib or erlotinib, given the common use of these agents. This work emphasizes not only the importance of avoiding alisertib but also suggests these patients would safely receive erlotinib. This study also emphasizes the different functional and signaling interactions of targeted inhibitors in distinct cellular contexts. Typically, AURKA inhibitors are used in therapeutic combinations with cytotoxic agents or other targeted agents (62): there is great interest in identifying productive therapeutic combinations. The positive interaction of alisertib and erlotinib in the context of oncogenic drivers (38), versus the opposing activity in the context of lesions in *Pkd1*, emphasizes the dynamic nature of signaling networks. Given the large number of comorbidities that are commonly experienced by cancer patients, including diabetes, cardiovascular disease, and other conditions that can affect cell signaling, there is clearly a need for further study.

## Conflict of Interest Statement

The authors declare that the research was conducted in the absence of any commercial or financial relationships that could be construed as a potential conflict of interest.
